# Spectrum analysis on quality requirements consideration in software design documents

**DOI:** 10.1186/2193-1801-2-310

**Published:** 2013-07-11

**Authors:** Haruhiko Kaiya, Masahiro Umemura, Shinpei Ogata, Kenji Kaijiri

**Affiliations:** Department of Computer Science, Shinshu University, 380-8553 Nagano, Japan

**Keywords:** Software engineering, Requirements analysis, Quality requirements, Software design document, Traceability

## Abstract

**Abstract:**

Software quality requirements defined in the requirements analysis stage should be implemented in the final products, such as source codes and system deployment. To guarantee this meta-requirement, quality requirements should be considered in the intermediate stages, such as the design stage or the architectural definition stage. We propose a novel method for checking whether quality requirements are considered in the design stage. In this method, a technique called “spectrum analysis for quality requirements” is applied not only to requirements specifications but also to design documents. The technique enables us to derive the spectrum of a document, and quality requirements considerations in the document are numerically represented in the spectrum. We can thus objectively identify whether the considerations of quality requirements in a requirements document are adapted to its design document. To validate the method, we applied it to commercial software systems with the help of a supporting tool, and we confirmed that the method worked well.

## Introduction

In the same way as functional requirements, quality requirements, such as security, usability, reliability, and efficiency should be defined at the requirements definition stage because these requirements are the dominant factor in development costs and efforts. How to define quality requirements completely and correctly is thus well studied (Kaiya et al. 2010a; Firesmith 2005; Zhang et al. 2008). However, we have no standard ways to confirm such quality requirements are adapted in upcoming software artifacts such as design documents or test cases. For functional requirements, traditional stepwise refinement techniques can be used for this purpose. At the last stage of software development, we can confirm whether quality requirements are adapted and implemented through testing. It is, however, too late to find incorrect or missing quality requirements in software implementation at this stage because the design and/or codes would need to be revised. We thus have to develop a method for validating quality requirements considerations in intermediate software artifacts, such as design documents.

In this paper, we propose and evaluate this method. We use a technique called “spectrum analysis for quality requirements” (Kaiya et al. 2008) to construct this method. The contributions of the method are as follows. First, software engineers can become aware of missing and/or incorrect quality requirements considerations in a design document even if such considerations are scattered over the document. Second, the software engineers can narrow down the parts of the design document containing the missing and/or incorrect quality requirements. Third, the method can be applied almost automatically because domain knowledge can be reused for spectrum analysis. Fourth, the method can be applied to most requirements and design documents because it does not require specific notations such as mathematical equations but that the documents be written in natural language. Fifth, the method can be applied to a wide range of development styles such as waterfall or spiral styles because it does not give any constraints on the ordering of software development. A CASE tool was developed for supporting analysts in performing this method.

The rest of this paper is organized as follows. In the section “Spectrum analysis”, we briefly introduce the “Spectrum analysis for software quality requirements” technique used to analyze software quality requirements in a document. We then propose the method for validating quality requirements considerations in a design document in the section “Method”. By using our method, we assume we can apply our spectrum analysis from the section “Spectrum analysis” to a design document. To apply this analysis to a design document efficiently, a supporting tool is crucial because the size of such a document is not so small. In the section “Supporting tool”, we explain the supporting tool, and how to use it about a design document. We then confirm the assumption that the spectrum analysis can be applied to a design document in the section “Preliminary evaluation” by comparing the result of an analysis performed by an expert in commercial systems with the result obtained by the method. In the section “Evaluation”, we show an evaluation to confirm that the method works well. We finally review related works, summarize our current results, and show future problems.

## Spectrum analysis

**Figure 1:**
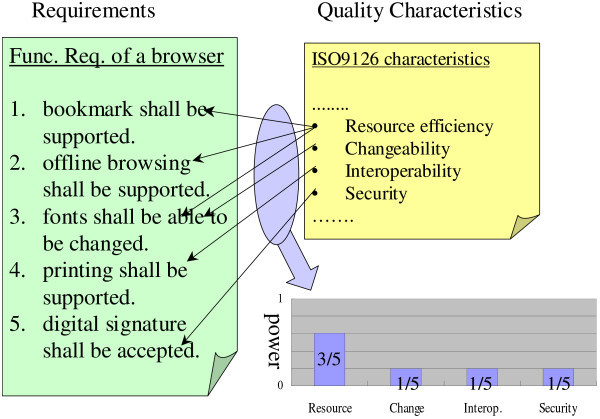
**Example of spectrum analysis for requirements.**

Spectrum analysis for quality requirements is one measurement technique for summarizing quality features scattered over a requirements document. The technique enables us to visualize quality characteristics in the same way as sound waves. We explain how to perform this analysis by using the example in Figure 1. In the figure, there is a list of requirements and a list of quality characteristics. These two lists are the inputs of the technique. An analyst decides the quality characteristics related to each requirement. For example, requirement #3 is related to both resource efficiency and changeability. After that, the analyst counts the number of requirements related to each quality characteristic. For example, resource efficiency is related to three out of five requirements. The counted number of each quality characteristic lets us know how often each quality characteristic is mentioned in a requirements document. We confirmed that the importance of each quality characteristic was reflected in the number (Kaiya et al. 2012). We call the number the “power” of a quality characteristic. To normalize the power, it is divided by the total number of requirements. For example, in this figure, resource efficiency is more important than the others because it is frequently mentioned in the list of requirements. We may give some weights to each requirement on the basis of the priority among the requirements. We may also reformat a requirements document (Ncube et al. 2007) if the document is highly structured like i* or KAOS goal models.

We can maintain the traceability links between the power of a quality requirement in a spectrum and requirements because the power is derived from the requirements. For example in Figure 1, the power of resource efficiency is derived from requirements #1, #2 and #3. When we want to know the reasons for the differences between two spectra, we simply trace the links mentioned above. The spectrum analysis for quality requirements only focuses on the type of quality requirements. It cannot handle the value and/or level of quality requirements. For example, it can detect whether a quality requirement related to resource efficiency is considered in a document. An example of such a quality requirement is “the number of users.” However, it cannot decide whether the number itself (e.g., 10, 100 or 10000 people) is correctly considered in a document. For this kind of decision on the value/level of quality requirements, specific methods are required for each type of quality requirements. This is one of the limitations of our spectrum analysis.

Establishing the relationships between requirements and quality characteristics in Figure 1 is not an easy task. To help, a term-characteristics map (TCM) was proposed, and its effectiveness was validated (Kaiya et al. 2010a). The TCM is a matrix between terms and quality characteristics defined in each problem domain. By looking up the TCM, the relationships in Figure 1 can be easily established because an analyst simply needs to check the occurrences of terms in each requirement. Developing a TCM is not easy task as well. We develop a TCM in a domain by gathering the results of a spectrum analysis. If sentences containing the term “xxx” are frequently related to a quality characteristic such as “security,” we develop a TCM containing the data that “xxx” is related to “security”. We empirically validated that the confidence of the TCM in each domain increases when the number of the results of the spectrum analysis increase (Kaiya et al. 2011).

## Method

In this section, we introduce the method for confirming quality requirements considerations in a design document by using spectrum analysis.

### Overview

**Figure 2:**
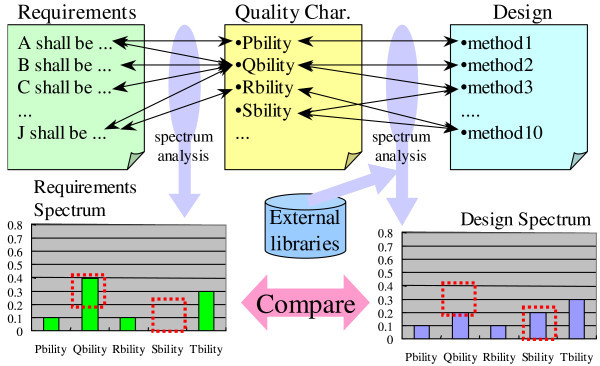
**Method for validating quality requirements considerations in a design document with spectrum analysis.**

Figure 2 shows the overview of the method. In the method, spectrum analysis is first applied to the requirements and design respectively. We then compare one spectrum with another to identify quality characteristics defined incorrectly and/or incompletely. In Figure 2, “Qbility” seems to be defined incompletely in design because the power of “Qbility” in the design spectrum is smaller than that in the requirements spectrum. “Sbility” seems to be defined incorrectly in the design because the power of “Sbility” in the design spectrum is larger than the power in the requirements. Because we can find methods (functions in desgin) related to each characteristic on the basis of the relationships between characteristics and methods, we can investigate the causes of such incompleteness and/or incorrectness. We explain each step in this method below.

### Spectrum analysis for requirements

The spectrum analysis for requirements is performed in accordance with the technique in the section “Spectrum analysis”.

### Spectrum analysis for design

There are several kinds of software design documents because there are several kinds of people who read them, such as project managers, programmers, architects, and testers. We focus on a design document for programmers in this paper. We thus regard the explanations for each method or function as a design document because programmers have to know the functionalities and roles of each method or function.

The spectrum analysis for design is also performed in accordance with the technique in the section “Spectrum analysis”. Instead of a sentence(s) for each requirement, a sentence(s) used for explaining each method is used for the spectrum analysis. We thus normalize the powers in a spectrum on the basis of the number of methods.

In addition, we have to revise the spectrum in accordance with external libraries such as application programmable interfaces (API) and/or frameworks. Both external organizations and the developer him/herself will provide such external libraries. Such a revision is performed as follows.

1. In accordance with the design description, external libraries to be used are selected.
2. For each library, quality characteristics supported by the library are identified. We call such characteristics “library-characteristics”. The documents for each library help us to identify them.
3. For each method, the libraries used in the method are identified. We then establish the relationships between the method and the library-characteristics of the libraries.

Aspect-oriented design (AOD) is currently out of the scope of this method. In the case of using AOD, the revision steps above cannot be applied because main routines do not know their required libraries (aspects), but the libraries know the routines that call the libraries. All aspects should be woven together before applying our method if AOD is used.

**Figure 3:**
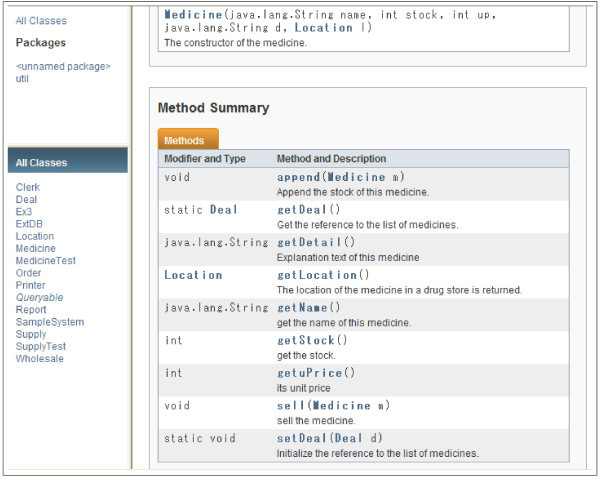
**Example of a design document.**

**Figure 4:**
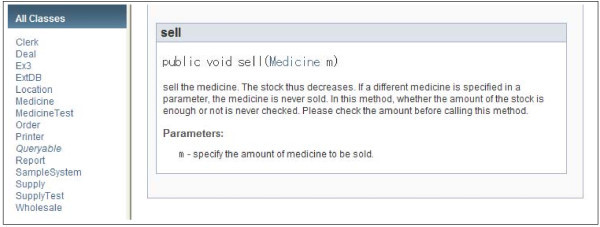
**Example of concrete description in a design document.**

We show an example of a design document for programmers in Figures 3 and 4 written in Javadoc (Oracle 2004). In this example, a supporting system for a drugstore is shown, and the system will be written in Java. In this design, 13 classes are defined except test classes. In Java, test classes are normally ended with “Test” because that is the common practice when using the unit test tool called “JUnit”. In Figure 3, methods in the class “Medicine” are listed with brief explanations. However, programmers cannot implement each method with only such brief explanations because such explanations contain only the functionality of the method. Designers thus have to give more detailed explanations for each method, as exemplified in Figure 4. In the figure, the method “sell” in the class “Medicine” is explained in detail. By reading this detailed explanation, programmers can correctly implement the method. In this detailed examination, programmers can know that the sell method does not require checking the amount of stock. These kinds of explanations avoid useless double checks for parameters that are required by the policy of “design by contract” (Meyer 1997). The spectrum analysis for the design is applied to this kind of detailed explanations. Terms and words that are used in the spectrum analysis depend on the contents of the TCM. In our evaluation, the same TCM is used in both the requirements and design documents.

### Comparison

We can simply visualize the spectra of both the requirements and design and identify the differences in order to find the causes of inconsistent and/or missing requirement considerations. When an engineer finds such causes, he or she modifies the design documents to remove them. We explain how to find such causes by using the example in Figure 2. As mentioned in the first sub-section of the section “Method”, we can find that “Qbility” seems to be defined incompletely in the design because its power in the design is smaller than that in the requirements. By examining the relationships at the top of the figure, methods except for methods 2 and 3 are suspected to cause the incompleteness because they are not related to “Qbility” in the figure. To remove such causes, an engineer has to establish more relationships between the quality characteristics and design than exist now. For “Sbility”, methods 3 and 10 are suspected to cause the incorrectness in the design because “Sbility” is not defined in the requirements but is defined in the design as shown in Figure 2. To remove such causes, the engineer has to establish less or no relationships between the quality characteristics and design than exist now.

Because a quality spectrum is a kind of vector, we use cosine similarity (cossim) to decide whether two spectra are similar to each other. Cosine similarity can be a metric for identifying the inconsistent and incomplete quality requirements considerations in design. The definition of cosine similarity between  and  is as follows.

**Figure 5:**
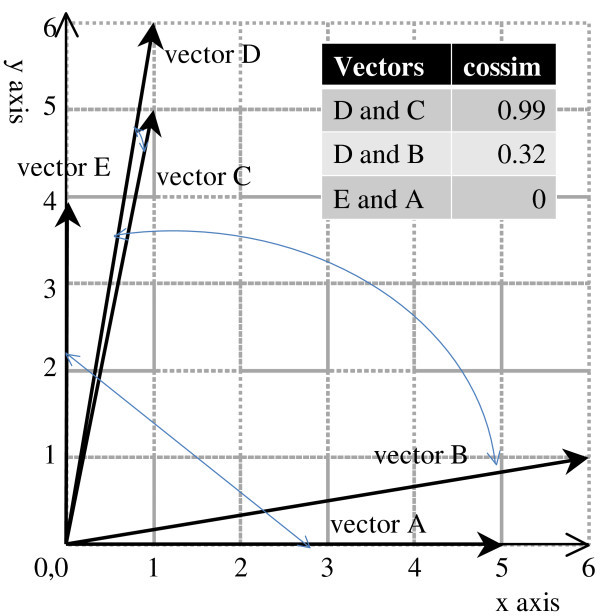
**Intuitive meaning of cosine similarity.**

When two vectors are completely the same, the value is 1. Because a quality spectrum never has a negative value in its vector, cosine similarity between two quality spectra varies from 0 to 1. Therefore, we may regard two quality spectra as similar if their cosine similarity is close to 1. In addition, cosine similarity does not take the length of the vector into account. Figure 5 shows an intuitive meaning of the cosine similarity with two-dimensional vectors. As show in the figure, two vectors D and C are almost the same as each other. The cosine similarity among them thus takes almost one (0.99). In comparison, vectors A and E make a right angle. As mentioned above, all values in a vector for our spectrum analysis take non-negative values. The direction of vector A is thus completely different from that of vector E. The cosine similarity between these vectors takes 0 by the definition above.

## Supporting tool

As mentioned in the section “Spectrum analysis”, the idea of the spectrum analysis is simple, but it is too dull for humans to perform the analysis manually. We thus developed a supporting tool to perform the spectrum analysis. Because the tool is designed to analyze natural language sentences, it can be applied to both requirements documents and design documents, such as Java docs.

### Functions and usage

The tool has the following three functions.

**Figure 6:**
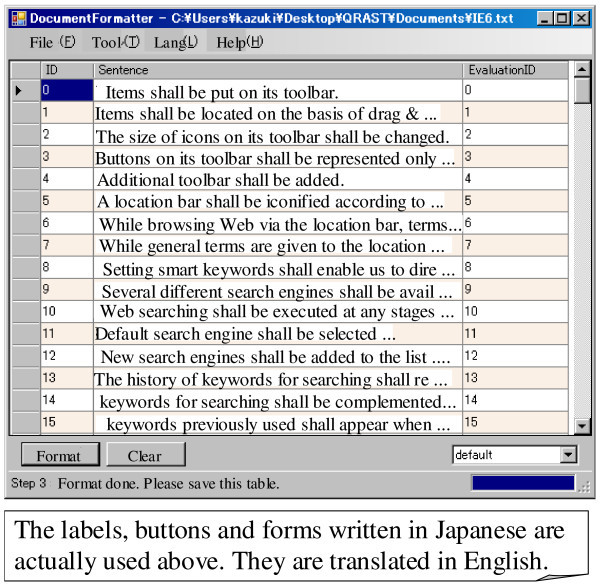
**Tool for formatting a document.**

1. Dividing a natural language document into a list of items:

An item is the unit for the requirements or design documents in our spectrum analysis. For example, a pair of comprised the name of a method and sentences used for its detailed explanation is a unit when a design document written in Java docs is analyzed. An item normally consists of one or several sentences. We can define several different rules for dividing a document into a list of items. We assume a text file contains the document and each line in the file contains one requirement sentence. In such a case, a document is divided into several items on the basis of the occurrences of line separators^a^. The number of the items is thus the same as the number of lines in the document. A document can be also divided into several items when a line begins with numbers such as 1, 2, or 3. A regular expression such as “^[0-9]” can be used for defining the rule for such division. Figure 6 shows an example of a GUI for this function. In this example, a normal rule that uses line seperators is used for the division. For design documents such as Java docs, we have to prepare a plain text containing the pairs.

**Figure 7:**
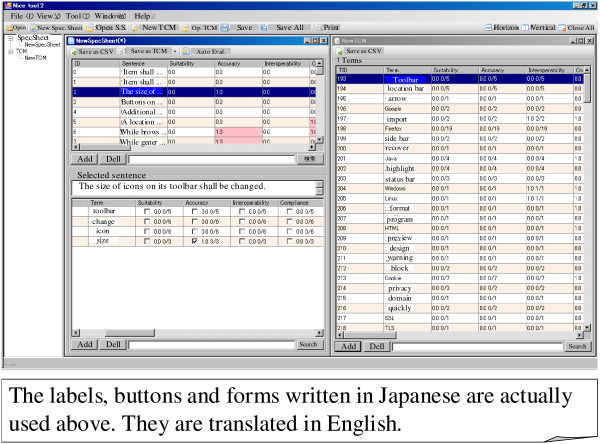
**Tool for performing spectrum analysis based on an existing TCM.**

2. Performing spectrum analysis on the basis of a list of items and TCM:

On the basis of a list of items generated by the first function and a predefined TCM, the spectrum analysis is performed automatically. A requirement analyst or someone else can develop a TCM manually with the help of a general spreadsheet or something like that. The predefined TCM can be prepared in such a way. The predefined TCM can be also prepared according to the step 3 below. Figure 7 shows an example of the result of this function. A sub-window labeled “NewTCM” in the right-hand side of this figure shows the predefined TCM. Each line of this sub-window corresponds to a term and its data in this TCM. The first row of this sub-window shows the internal identifier of a term (TID). The second row shows the term itself. The third, fourth, and the other rows show the quality characteristics, such as suitability, accuracy, and interoperability. Each cell except those in the first and second rows contains numbers such as “0.0 0/5”. These values show the probability for whether a term is related to a quality characteristic. When its form is “p x/y”, the value of p indicates the probability, and the value is derived by dividing x by y. The value of y is the number of items, each of which contains a term in the predefined TCM. The value of x is the number of items, each of which contains a term and is semantically related to the quality characteristic in the predefined TCM. For example, a cell for the term “Windows” (its TID is 204) and “Interoperability” contains “1.0 1/1”. The meaning of the value is as follows. The term “Windows” occurred in an item during the past analyses, and the item was semantically related to “Interoperability.” Therefore, we may assume a new item containing the term “Windows” will be 100 percent (i.e., 1.0) related to “Interoperability”. A sub-window labeled “NewSpecSheet” in the left-hand side of the Figure 7 shows the results of the spectrum analysis. The top area of the sub-window shows the list of items in Figure 6. In this example, the second item is selected in the figure. The bottom area of the sub-window shows four terms automatically picked-up from the second item. For each term, probabilities for whether a term is related to each quality characteristic are automatically looked up in the TCM. The looked-up probabilities are also shown in the bottom area. An analyst may accept the probabilities, and decide that an item is related to specific quality characteristics. He or she may also ignore the probabilities, and make another decision.

3. Updating the TCM on the basis of the current result of the spectrum analysis:

On the basis of the current results of the spectrum analysis, the current TCM may be updated. As mentioned in the section “Spectrum analysis”, the confidence of the TCM increases when the historical data of the TCM usage is gathered. This feature was validated through our experiment (Kaiya et al. 2011). When no current TCM exists, a new TCM is created on the basis of only the results of a current analysis.

### Implementation

The tool was implemented in C#, and .NET Framework 2.0 was used. For the GUI, Windows forms were used, and SQLite (Hipp et al. 2013) was used for data processing. Because SQLite simply uses a file as a database, the portability of this tool is very good, i.e., we do not have to setup the database server in addition to our tool. Because the tool was designed and implemented for processing Japanese sentences and there are no explicit separators between words such as spaces or tabs in a Japanese sentence, we had to split a sentence into terms and words on the basis of the meaning of the sentence. We used a Japanese morphological analyzer called “Chasen” (Nara Institute of Science and Technology 2011) to identify terms and words in a sentence automatically. For the performance, it took about 3 seconds to process about 200 items under a TCM with about 200 terms. We used a PC with a 1.73 MHz Celeron M processor.

## Preliminary evaluation

### Overview of preliminary evaluation

The spectrum analysis on requirements specifications was already evaluated (Kaiya et al.2010a), so we can assume that the derived spectrum is equivalent to the intuitive evaluation of a requirements specification performed by an expert in the application area. The objective of this preliminary evaluation is thus to confirm the same assumption for design documents. We evaluate the design spectrum before it is revised by using external libraries because we want to know whether explanation sentences for each method can be used for the spectrum analysis.

The steps in this evaluation are as follows.

1. We ask an expert to identify the quality characteristics related to each method in a design document.
2. On the basis of this identification, we derive a spectrum by using the spectrum analysis in the section “Spectrum analysis”. We call the spectrum an “expert spectrum.”
3. We prepare TCMs for each application domain.
4. By using the TCM, we automatically derive a spectrum of the document. We call the spectrum a “TCM spectrum.”
5. We compare these two spectra because we expect they are almost the same.

### Systems to be used in this evaluation

Here, we introduce the systems to be used in this evaluation. An overview of the systems is shown in Table 1. For ease, we call them “Movie Player” or “PDF Viewer” instead of their original names. Both systems are applications running on Mac OS. They are commercial software because they are sold on the Mac App Store (Umemura 2011,2012). With the help of the developer of these applications, we can know their development data, shown in Table 1, even though they are not open-source software. The developer said requirements were correctly adapted to the design document in each system.

**Table 1:** **Overview of systems to be analyzed**

Type	Name	LOC	NOM	NOC	NOR
Movie Player	Meteoroid	11277	167	21	61
PDF Viewer	Dioretsa	4640	84	7	60

Acronyms are lines of codes excluding comments (LOC), number of methods (NOM), number of classes (NOC) and number of requirements (NOR).

As mentioned in the previous section, we focus on the design document for programmers. Programmers have to know the functionalities and roles of each method in each class. We thus regard the explanations for each method as design documents. Because the systems are written in Objective-C, the design documents are written in HeaderDoc (Apple Inc. 2013), which is similar to Javadoc. In addition to the standard API and frameworks, an external API is used only in the PDF Viewer (Dioretsa). This is one of the reasons the lines of code (LOC) for the PDF Viewer are smaller than those for the Movie Player, as shown in Table 1, even though the number of requirements (NOR) is almost the same.

### Results and discussion

**Figure 8:**
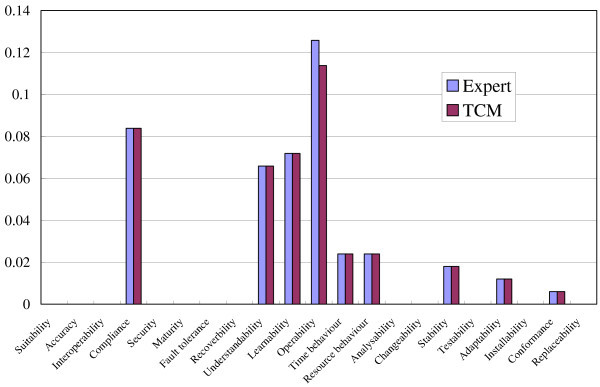
**Expert spectrum and TCM spectrum for Movie Player.**

**Figure 9:**
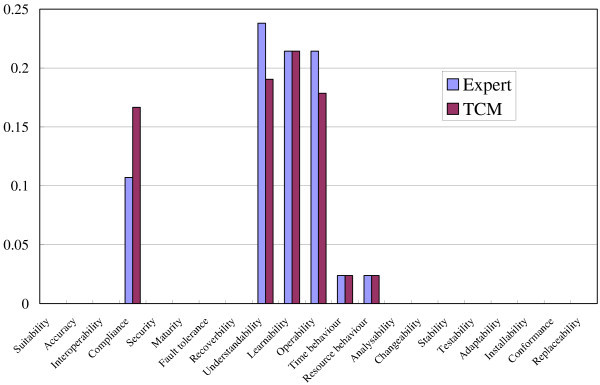
**Expert spectrum and TCM spectrum for PDF Viewer.**

We used quality characteristics defined in the ISO 9126 standard (International Standard ISO/IEC 9126 1991) as the categorization of quality requirements. Figure 8 shows the result of a comparison between the expert spectrum and the TCM spectrum for the Movie Player. We regard two spectra is the same when their cosine similarity is bigger than 0.95. As shown in the figure, two spectra were almost the same, and their cosine similarity was 0.9987. The result for the PDF Viewer is shown in Figure 9, and the cosine similarity was also 0.9784. We may thus conclude that we can apply the spectrum analysis in the section “Spectrum analysis” to the design documents such as HeaderDoc.

## Evaluation

### Overview of the evaluation

The objective of this evaluation was to confirm whether the method in the section “Method” works well. In other words, we wanted to confirm that the quality requirements defined in a requirements specification were correctly adopted in the design document. We then applied the method to systems introduced in the section “Preliminary evaluation”, and analyzed the results. As mentioned in the section, quality requirements are correctly adopted in the design document in each system. We thus expect the two spectra for the requirements and design to be almost the same. In the same way as the preliminary evaluation, we also used quality characteristics defined in the ISO 9126 standard (International Standard ISO/IEC 9126 1991) as the categorization of quality requirements. The standard contains 21 characteristics as mentioned in the results below.

### Results

**Figure 10:**
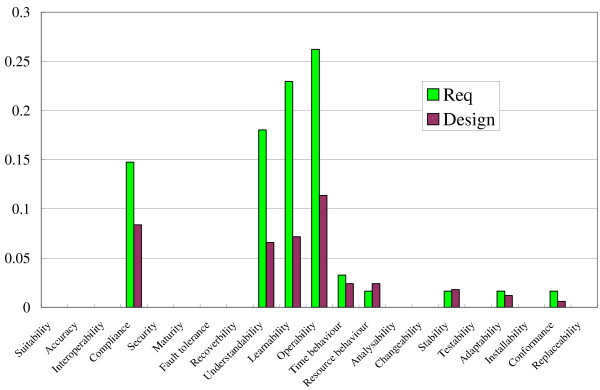
**Result of method for Movie Player.**

Figure 10 shows the result of the method for the Movie Player. Because a spectrum is a 21-demensional vector for the 21 quality characteristics and the method outputs two spectra for the requirements and design, these two spectra are visualized as bar charts in the figure. The spectrum for the design was not revised in accordance with the external libraries because no external libraries were used in the Movie Player. Although the shapes of the graphs were not similar, the cosine similarity of these two spectra was about 0.9722. The result shows that the two spectra (vectors) pointed in a similar direction, but the lengths of the vectors differed.

**Figure 11:**
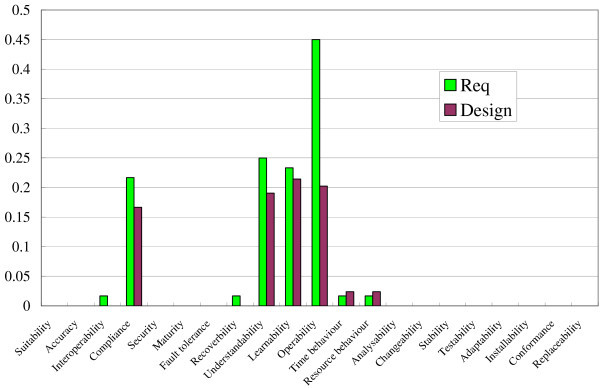
**Result of method for PDF Viewer.**

Figure 11 shows the result of the method for the PDF Viewer. Because external libraries were used in the system as mentioned in the section “Preliminary evaluation”, the spectrum for the design was revised in accordance with them. The cosine similarity of these two spectra was about 0.9543. The result shows that the two spectra (vectors) pointed in a similar direction.

### Discussion

Judging the results above, the methods seemed to works well, although the lengths of vectors (spectra) were not similar but pointed in a similar direction. Because the lengths were not similar, there is a possibility that all quality characteristics were equally diminished in the design document. However, the possibility is too unlikely to occur because more than 20 characteristics were used in the spectrum. A more plausible cause for the difference of the lengths is the way the normal form of each spectrum was derived. As mentioned in the section “Method”, each value in a vector (a spectrum) for a requirements document is not the same as the number of requirements related to each quality characteristic but the same as the number of requirements divided by the number of all requirements. Because each requirements document contains a different number of requirements, we have to normalize each spectrum in this way. In the same way as the requirements document, a design document is normalized in accordance with the number of methods. This idea will cause the difference of the spectra lengths.

One idea to solve this problem is to normalize the spectrum of design by using the number of requirements. The quality characteristics are the concepts at the requirements stage even when they embedded in design documents, test cases, or codes. The lengths of the spectra become almost the same when we apply this idea in the systems. However, our method only focuses on the similarity of the direction among the spectra, so we do not have to solve this problem now. The problem has a bad effect only on the visual effects, as shown in Figures 10 and 11, but it never affects the results of cosine similarity.

**Figure 12:**
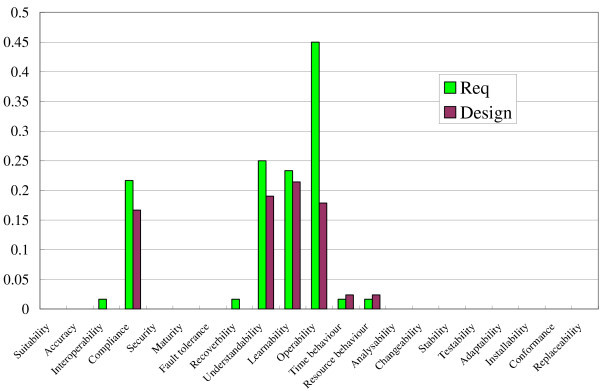
**Result of method for PDF Viewer before the revision based on the external libraries.**

We discuss the effectiveness of the revision caused by the external libraries. We applied the method without the revision to the PDF Viewer. The result is shown in Figure 12, and we obtained 0.9374 as the cosine similarity between the requirements and design. Because the cosine similarity was 0.9543 in the method with the revision as mentioned above, the revision seemed to improve the results. We cannot perform the same test for the Movie Player because it does not use the external libraries.

Finally, we mention the threats to the validity of this evaluation. Threats to internal validity are related to whether the data comparison is properly measured. Because both requirements and design spectra were derived systematically in accordance with the steps in our method, two spectra were properly measured. We do not have to worry about the learning effect because there are few steps for subjective decision in our method. We thus conclude that there are no threats to internal validity. Threats to external validity are related to the generalizability of the results. Although commercial software systems were used in this evaluation, they were developed in Objective-C, which is a little bit different from other languages such as java or C#. In this sense, a threat to external validity can exist. However, there are no significant differences for the requirements and design documents because the requirements are written in natural language sentences and the design documents are written in HeaderDoc, which is similar to Javadoc documents. In this sense, there are no threats to external validity. Construct validity is related to the measurement of data. As mentioned above, there is a problem with the length of a design spectrum because the spectrum is normalized on the basis of the number of methods. In this sense, there is a threat to construct validity. We however do not mind this point because a metric cosine similarity focuses not on the length of the spectrum but only on its direction. There is a threat to conclusion validity because we cannot perform statistical analysis only with two treatments.

## Related works

Software quality requirements are widely focused on in the field of software, and we can find a special magazine issue of IEEE Software published in 2008 (Blaine and Cleland-Huang 2008). In the issue, the importance and challenges of software quality requirements were summarized, and one of the challenges is measurement and traceability for software quality requirements. In this section, we briefly review research on this measurement and traceability to clarify the importance of software quality requirements. We use non-functional requirements (NFRs) as a synonym for quality requirements even though NFR contains more things than do quality requirements (Blaine and Cleland-Huang 2008; Glinz 2008).

First, we focus on *traceability links* among different kinds of software engineering artifacts. Managing explicit links among different artifacts is a normal idea. For example, links between a section in a requirements document and classes and packages in design documents help us to find the impacts of changes on design.

However, maintaining such links takes a lot of effort in general. To mitigate such effort, several kinds of ideas are proposed. Lucia et al. used information retrieval techniques to manage such links (Lucia et al. 2009). Mandelin et al. used a probability model to do that (Mandelin et al. 2005). Ratanotayanon et al. used the differences between different versions of artifacts to manage traceability links efficiently (Ratanotayanon et al. 2009). Lopez et al. used the techniques of natural language processing (NLP) and machine learning (ML) to trace quality requirements to architecture (Gokyer et al. 2008). Model-driven approaches can enable us maintain traceability automatically. Alebrahim et al. proposed a method to derive software architectures from quality requirements (Alebrahim et al. 2011). In the method, problem frames (Jackson 2000) and its UML profile was used for specifying functional and quality requirements. They also used patterns for deriving architecture. Although the method seems to work well for maintaining traceability, we have to use specific and normally complex notations.

Second, we focus on *a central model* shared by software engineering artifacts. By using this model, we can easily trace an artifact to another via the model. Jane et al. proposed a method called “Goal Centric Traceability” for quality requirements (Cleland-Huang 2005; Cleland-Huang et al. 2008). In the method, a goal model plays the role of a central model. The thesaurus and ontology are popular notations for a central model. Daneva et al. proposed an ontology for NFRs (2009; Kassab et al. 2008). However, how to make links between ontology and software artifacts was not mentioned. Saeki et al. used a domain ontology for traceability between documents and source codes (Yoshikawa et al. 2009).

Kaiya *et al* proposed another idea for traceability called “projection traceability” (Kaiya *et al.*2010b*), and the method proposed in this paper is based on this idea. In that paper (Kaiya et al.*2010b*), the traceability between requirements and codes is analyzed, but the execution tests can be used instead of this analysis because the codes can be executable. In comparison, the method in this paper is about the traceability between requirements and design, and it helps the software developers find missing and/or incorrect quality requirements considerations earlier than with the analysis in (Kaiya et al.*2010b).

Finally, we briefly review research on measuring quality requirements. One famous catalog for software quality requirements is the ISO9126 standard (International Standard ISO/IEC 9126-1 2001*), which contains about 20 subcharacteristics such as accuracy, and reliability. Washizaki et al. provided measurement methods that use the usual metrics on source codes and design diagrams such as lines of codes (LOC) and cyclomatic complexity (CC) for each subcharacteristic (Washizaki et al.*^2008^*). Jane et al. proposed a method for detecting and categorizing NFRs contained in a document by using information retrieval (IR) and NLP techniques. (Cleland-Huang et al.*^2006^*). To count and normalize the number of NFRs in a document, we can visualize the distribution of NFRs. Kaiya et al. proposed a technique for summarizing such a distribution and visualizing it on the basis of a metaphor of spectrum analysis in optics (Kaiya et al.*^2009^*). They used the technique to identify domain specific commonality by directly comparing one spectrum of a system to another. Whether a requirement is related to a quality requirement is decided on the basis of the occurrences of terms, which characterize the quality requirement in the study. Measuring quality requirements is used for prioritizing requirements (Otero et al.*^2010^*), but how to establish relationships between quality requirements (a quality feature in the study) is not explained in the study. Because quality requirements qualify functional requirements, measuring and predicting quality requirements on the basis of the content of each functional requirement is a natural idea. Kaiya et al. developed such an idea by using semi-formal functional requirement notation (Kaiya and Ohnishi*^2011^*). They also used a machine learning technique to automate the prediction and measurement (Tanaka et al.*^2012^). This kind of research is useful for tracing quality requirements indirectly.

## Conclusion

In this paper, we proposed the method for validating quality requirements considerations in a design document. We also proposed a supporting tool for the method. Because the method uses spectrum analysis for quality requirements, it does not give any constraints on design notations and activities. Through an evaluation on commercial software systems, we confirmed the method works well.

Currently, we have no integrated CASE tools for supporting design activities and design analysis based on this method. Our current tool analyzes only documents on the basis of our spectrum analysis, so analysts have to manually modify these documents if problems are found. One important goal in the future is to develop an integrated CASE tool for supporting both design descriptions and analyses for software development.

## Endnote

^a^ Line separators depend on the kinds of operating system. For example, a line separator in UNIX is normally ∖*n*, while one in Windows is ∖*r*∖*n*.
